# The Role of Orthodontic Treatment in Preparing the Potential Implant Prosthetic Space for Implant-Supported Single-Tooth Restorations

**DOI:** 10.3390/medicina62030580

**Published:** 2026-03-19

**Authors:** Amelia Smaranda Roșianu, Stelian Mihai Sever Petrescu, Ionela Elisabeta Staicu, Mihaela Ionescu, Cosmin Mihai Mirițoiu, Sanda Mihaela Popescu

**Affiliations:** 1 Department of Oral Rehabilitation, University of Medicine and Pharmacy of Craiova, 200349 Craiova, Romania; amelia.popescu@umfcv.ro (A.S.R.); sanda.popescu@umfcv.ro (S.M.P.); 2 Department of Orthodontics, University of Medicine and Pharmacy of Craiova, 200349 Craiova, Romania; stelian.petrescu@umfcv.ro; 3 Department of Medical Informatics and Biostatistics, University of Medicine and Pharmacy of Craiova, 200349 Craiova, Romania; 4 Department of Applied Mechanics and Civil Constructions, Faculty of Mechanics, University of Craiova, 200585 Craiova, Romania; cosmin.miritoiu@edu.ucv.ro

**Keywords:** potential implant prosthetic space, fixed appliances, Invisalign, Spark, duration of orthodontic treatment

## Abstract

*Background and Objectives*: When a single missing tooth must be replaced, the best solution is the placement of an implant. In adults, most of the time the space for implant is totally or partially closed due to the shift in the adjacent teeth. The objective of the study was to describe the clinical parameters, treatment choices, and outcomes associated with orthodontic space opening for single-tooth implants in various treatment solutions, as well as to determine their influence on the variation in the dimension of the edentulous space. *Materials and Methods*: An observational prospective cohort study was designed in which patients with a single missing tooth were selected to be included in the study. After the clinical examination two groups were formed: patients who opted for fixed orthodontic treatment (metallic or sapphire) to open space for implant and patients who opted for alignment to achieve this (with Invisalign or Spark). All subjects received orthodontic treatment. A dental chart was created for each patient which included demographics, clinical data, orthopantomography (OPG), profile cephalograms, and photographs. The potential implant prosthetic space was measured during orthodontic treatment to observe the space dimension evolution in time. *Results*: In total, 97 patients were included in the study, 60 women and 37 men, with ages between 14 and 60 years. Edentulous spaces dimensions were opened from 1–4 mm to 5–6 mm (39.18% patients), 6–8 mm (48.45%), and >8 mm (12.37%). Both types of orthodontic treatments were effective in opening the potential prosthetic space for implant. *Conclusions*: Large prosthetic spaces and older edentulism tend to require longer treatments. Older patients had experienced edentulism for a longer period, indicating a correlation between age and the duration of tooth loss. Metal fixed orthodontic appliances were used in exceedingly long treatments, while aligners/sapphire brackets were used in short–medium durations. For the study group, fixed appliances and aligners proved to be effective in opening the space for future implants.

## 1. Introduction

In the practice of dentistry, it is common for patients who require dental implants to have a deficit of potential prosthetic space, which is why the intervention of an orthodontist is requested before the implant is inserted [1]. Creating space for a prosthetic implant represents a challenge for both the orthodontist and the prosthodontist [2]. The choice of method for space creation must consider multiple factors and requires close interdisciplinary collaboration [3,4]. It is important to highlight the multidisciplinary aspects of oral rehabilitation treatment involving specialties such as periodontics, dental implant, orthodontics, and prosthodontics [5]. Orthodontic movement of migrated teeth in adults has an important place in complex oral rehabilitation plan. Although the biological principles governing tooth movement are identical, the biomechanics differ between fixed appliances and clear aligner systems due to differences in force delivery and anchorage control. In fixed appliance therapy, space opening is achieved through controlled mechanical force systems generated by brackets and archwires. After initial alignment with light round nickel–titanium wires, space is typically created using compressed open-coil springs (nickel–titanium) placed between brackets. These springs deliver continuous light forces that separate adjacent teeth [6]. As treatment progresses, rectangular stainless steel archwires are introduced to enhance torque and root control, ensuring bodily movement rather than uncontrolled tipping. Anchorage management is critical, as space opening inherently produces reciprocal forces. Reinforcement is achieved using temporary anchorage devices (TADs) that provide skeletal support [7,8]. Fixed appliances therefore offer a high degree of three-dimensional control, particularly with respect to root positioning and torque expression [9]. In clear aligner therapy, space opening is digitally planned and executed through staged tooth movement. The desired space is incorporated into the virtual treatment setup and movement is distributed incrementally across a series of aligners, typically allowing 0.1–0.3 mm of movement per stage [10]. The thermoplastic aligners apply forces over the crown surfaces of the teeth, and auxiliaries are often required to improve force efficiency [11]. Composite attachments enhance retention and facilitate specific movements such as bodily translation or root torque. Power ridges are frequently incorporated to improve anterior torque control during space development [12,13]. Anchorage is managed digitally through controlled staging of reciprocal movements, and interarch elastics are sometimes prescribed when additional anchorage reinforcement is required. In anterior regions, virtual pontics are included within the aligners to maintain aesthetics while space is being developed. Although aligner systems have become increasingly predictable, significant bodily movements and root control can be more technique-sensitive and may require refinement stages [14]. Furthermore, treatment success is highly dependent on patient compliance with the prescribed wear protocols [10,14].

Because orthodontic treatment with braces or clear aligners may have adverse effects such as pain, root resorption, and periodontal problems, clear aligners may be a better option for patients with higher periodontal risk or difficulties in maintaining oral hygiene [15]. Fixed orthodontic appliances remain effective for complex cases but tend to increase plaque buildup and gingival recession, requiring strict hygiene measures. Overall, the choice between aligners and fixed appliances should be guided by individual factors such as age, motivation, and periodontal condition to ensure the best periodontal outcomes. Despite expectations, the clear aligner system did not demonstrate better “periodontal performance” than conventional devices [16]. This was probably because the coverage, all day, of dental surfaces, can lead to the accumulation of dental plaque resulting in inflammation [16]. Furthermore, the treatment may also affect the patient’s chewing and speech functions, leading to changes in their quality of life [17].

Orthodontic treatment in partially edentulous patients is challenging because adjacent teeth often tip or over erupt, reducing the space needed for implant placement. Orthodontically reopening these partially or fully closed spaces is essential for successful prosthetic rehabilitation and for restoring the patient’s function and aesthetics [18]. Thus, when a midline deviation is associated with an asymmetric dental arch and the absence of a molar on the narrower side, maintaining or creating the edentulous space and performing arch expansion is indicated rather than closing the space in order to correct transverse discrepancies and midline deviation [19]. Close collaboration between the orthodontist and the general dentist is crucial to determine the appropriate prosthetic space and ensure proper integration of the future implant [20]. Therefore, it is of interest to evaluate the behavior of clear aligners compared to fixed orthodontic therapy in the retraction of anterior teeth, using finite element analysis. The fixed appliance shows greater anterior torque control and improved safety for the posterior anchorage teeth comparative to clear transparent aligners [21,22].

Pre-prosthetic orthodontics also play an especially significant role in treating oligodontia. Maxillary lateral incisors are congenitally missing in 1–2% of the population. Their management requires an interdisciplinary approach combining orthodontic, restorative, and implant prosthetic treatments, often with adjunctive orthodontics to create or redistribute space and achieve an optimal aesthetic result [23]. Treatment plans can include orthodontic space opening or closure before prosthetic therapy [24,25]. Research in this area has demonstrated that orthodontic treatment is particularly important in creating the space for the future placement of an implant [26,27,28]. In these cases, anchorage is essential, especially when it is necessary to upright the teeth adjacent to the edentulous space. Proffit WR et al. [29] defined it as the resistance to unwanted tooth movement.

Furthermore, digital instruments play a key role in creating the space for implants through orthodontic treatment. Therefore, Bianchi et al. [2] emphasized that integrating multiple imaging sources (such as CBCT scans and 3D digital dental models) is essential for accurate planning and management of implant surgery combined with orthodontic treatment.

In a study highlighting the importance of interdisciplinary collaboration in planning implant-supported restorations for young patients, Kokich et al. [30] analyzed the key aspects that must be addressed to ensure the aesthetic outcome of implant restoration. Creating sufficient space for implant by fixed or mobile appliances plays a fundamental role in pre-prosthetic orthodontics.

The aim of this study is to describe the clinical parameters, treatment choices, and outcomes associated with orthodontic space opening for single-tooth implants in various treatment solutions, and determine their influence on the variation in the dimension of the edentulous space.

## 2. Materials and Methods

### 2.1. Study Design, Ethics, Sample Selection

The design of the prospective clinical study was an observational prospective cohort study, with a non-randomized cohort where treatment was chosen by the patient/clinician. The study included patients with single-tooth edentulism referred to the Orthodontics Clinic for opening the space necessary for dental implants. These patients came from the Oral Rehabilitation Clinic of University of Medicine and Pharmacy of Craiova, where they presented for oral rehabilitation treatment. Each time a potential implant prosthetic space was diminished and an implant was planned for rehabilitation, the patient was referred to the orthodontist for treatment. The study followed the evolution of patients from the moment they presented to the orthodontist, during the progress of the treatment. In order to determine the minimum number of participants, a sample size calculation was performed using G*Power 3.1.9.7 (from Heinrich Heine University Düsseldorf, Düsseldorf, Germany) considering a significance level α of 0.05, a power 1-β equal to 0.8, and a medium effect size value (since there are very few data available in the literature, and with an awareness of practical significance), thus ending up with a study lot of 94 participants.

The study was registered on ISRCTN, under the title of *How braces can make space for a dental implant to replace a missing tooth: a clinical study*, reference 48466, ISRCTN68311617.

The study complied with the Declaration of Helsinki, with each patient giving their consent to participate in the study and to undergo dental and orthodontic treatment. For all participants under the age of 18, written informed consent was obtained from parents or legal guardians.

The study was approved by the Ethics Committee of the University of Medicine and Pharmacy (no. 156/30 August 2023).

*Inclusion criteria*

• Patients with single-tooth edentulism, with the possibility of future implant prosthetic restoration;
• Patients with general conditions that do not contraindicate orthodontic treatment (ASA I or II);
• Patients with stable occlusion;
• Patients who have accepted orthodontic treatment.

*Exclusion criteria*

• Patients with serious general conditions, ASA grade above ASA II;
• Patients with extensive partial edentulism, total edentulism;
• Patients with unstable occlusion;
• Patients with skeletal anomalies requiring complex orthognathic and orthopedic treatments.

### 2.2. Intervention

The study included patients with single-tooth edentulism for whom the neighboring teeth presented position changes that created occlusal imbalances. For adult patients over 18 years of age, the option of implant prosthetic treatment was taken into consideration, requiring orthodontic management of the edentulous space. In addition to adult patients, the study also included adolescents, with ages between 14 and 18 years old, who required management of the edentulous space to position an implant in adulthood. For adolescent patients, the presence of a single-tooth edentulous space required consideration of orthodontic treatment to maintain or close the edentulous space, to prevent tooth migrations that would create malocclusions.

The patients included in the study were allocated into two lots, each with two sub-lots, depending on the type of orthodontic treatment performed. The two groups were: the group with fixed orthodontic treatment and the group with clear aligners. The allocation of patients in each group and subgroup was made according to the following criteria:

1. Severity of cases:
  a. cases that required greater force to realign molars were assigned to the bimaxillary fixed metal appliance subgroup; at the same time, cases that presented dentoalveolar disharmony with pronounced crowding greater than 6 mm were assigned to this subgroup;
  b. cases that required greater force for molar realignment but presented mild dentoalveolar disharmony, with crowding less than 6 mm, were assigned to the sapphire appliance sub-group;
  c. mild and moderate cases that did not require high forces for molar realignment were assigned to the clear alignment, Invisalign, or Spark sub-group.
2. Aesthetic criterion: Participants who preferred an aesthetic appliance opted for clear aligners.
3. The financial aspect was also very important: some patients preferred a fixed appliance because it was cheaper than the clear aligners.

The group with fixed orthodontic treatment had two subgroups: the subgroup with metal brackets (for which fixed bimaxillary metal appliance was used with tubes for molars 6 or 7 and round followed by rectangular springs, starting from small forces—0.14 NiTi up to 19 × 25 steel rectangular spring) and the subgroup with sapphire bracket (for which fixed bimaxillary sapphire appliance was used with tubes for molars 6 or 7 and round and rectangular springs, starting from small forces—0.14 NiTi up to 19 × 25 rectangular steel spring). The group with clear aligners had two subgroups: the subgroup with Invisalign clear aligner (for which it was used a set-up of orthodontic aligners customized according to the severity of the clinical case in question) and the subgroup with clear aligner Spark type. In the fixed appliance group, treatment was performed using a 0.022″ slot pre-adjusted edgewise appliance (straight-wire) system. Leveling and alignment were achieved with sequential nickel–titanium archwires, followed by rigid 0.019″ × 0.025″ stainless steel archwires for space opening. Compressed nickel–titanium open-coil springs were used to generate approximately 100–150 g of force per site, verified with a calibrated force gauge, and were reactivated at 4–6 week intervals. Temporary anchorage devices (TADs) were employed in cases requiring maximum anchorage control. In the clear aligner group, space opening was digitally staged with programmed increments of 0.1–0.3 mm per aligner. Patients were instructed to wear aligners for a minimum of 22 h per day, with changes every 10 days. Optimized composite attachments were placed on adjacent teeth to facilitate bodily movement and torque control, and power ridges were incorporated in anterior cases when additional incisor torque expression was required. Interproximal reduction was performed only when necessary to relieve crowding, and interarch elastics were prescribed when additional anchorage reinforcement was indicated. Refinement aligners were used when clinically necessary to achieve the planned prosthetic space.

Patients with mild dental abnormalities received treatment with aligners for 1 or 2 years, depending on the severity of the abnormality. Patient compliance was vital in these cases.

One orthodontist was involved in the evaluation of patients, their diagnosis and treatments, and the second orthodontist was involved in extracting data from the orthodontic charts. For the examiner orthodontist, calibration was performed on a set of 10 randomly selected participants. Intra-rater reliability was assessed using the intraclass correlation coefficient (ICC), computed based on a two-way mixed-effects model for absolute agreement, resulting in an excellent agreement (0.951).

The estimated duration of orthodontic treatment was 2–2.5 years. The primary outcome has been defined as the change in prosthetic space (mm), calculated as the difference between post-treatment and baseline mesiodistal space at the site of the missing tooth. The final dimension of prosthetic space for implants was counted from 5 mm, since the smallest implants have diameters under 3 mm (example 2.1 mm), and between the implant and tooth there should be a space of 1.5 mm. Subsequent implant placement was not evaluated, since this study followed only the orthodontic phase of the rehabilitation treatment.

At baseline, the orthodontic parameters registered were the occlusion Angle class type, position of the interincisal line relative to the mid-sagittal line, type of edentulism, location of edentulism, type of missing tooth, age of edentulism, size of the edentulous space, and type of orthodontic treatment. The duration of orthodontic treatment was measured using patient clinical records at the end of treatment.

The size of the potential prosthetic space and its changes were measured using orthopantomograms (OPGs) and intraoral compass at baseline and during treatment.

The study began in August 2023 and finished in November 2025. Date of first enrolment was 1 October 2023, while date of final enrolment was 1 December 2023.

### 2.3. Data Collection

For each patient presented at the Orthodontics Clinic, a dental file was completed, which included demographic data, data extracted from the clinical examination, OPG radiographs, and photographs. At the beginning of the study, the length of the edentulous space in mm was measured with a caliper at the cervical midpoint of approximal face of teeth situated mesial and distal from the edentulous space. Edentulous spaces with dimensions of 0 ≤ 1 mm were closed.

Potential prosthetic space (edentulous space) evolution was assessed using digital measurements obtained from standardized orthopantomograms (OPGs) and verified on the plaster models using a digital caliper. The exact landmarks used for measurement were from the distal midpoint of the cementoenamel junction (CEJ) of the tooth; from the mesial end of the edentulous space to the mesial midpoint of the CEJ of the tooth; and from the distal end of the edentulous space. The measurements were done by a single person, an orthodontist, at the beginning of the study and at the end of the follow-up period (variable from 1 to 1.5 years).

Radiographic measurements were calibrated according to the manufacturer’s magnification factor, and measurements were restricted to the mesiodistal dimensions in the anterior region to minimize distortion bias [31,32,33]. One examiner performed all measurements. Prior to data collection, examiner calibration was conducted using repeated measurements of 10 randomly selected cases at a two-week interval. Intra-examiner reliability was evaluated using intraclass correlation coefficients (ICC), with values greater than 0.90 considered indicative of excellent reproducibility. Any discrepancy exceeding 0.5 mm was reassessed to reach consensus. To minimize measurement bias, all records were anonymized and coded, and the examiner was blinded to the treatment group allocation and treatment stage during analysis.

### 2.4. Variables

The variables studied were:

1. Demographic data: Gender, age, and background.
2. Data extracted from the clinical examination: Angle class of malocclusion, position of the interincisal line relative to the mid-sagittal line, type of edentulism, location of edentulism, type of missing tooth, duration of tooth loss, size of edentulous space, type of orthodontic treatment, and duration of orthodontic treatment.
3. Size of the potential prosthetic space before and after treatment.

### 2.5. Statistical Analysis

The data extracted from the dental files were entered into an Excel spreadsheet (Microsoft Excel 365, Microsoft Corporation, Redmond, WA, USA) and statistical analysis was performed in Microsoft Excel 365 (Microsoft Corporation, Redmond, WA, USA) and SPSS (IBM SPSS Statistics 28, IBM Corporation, Armonk, NY, USA).

The associations between categorical data were analyzed using Chi-square χ^2^ and Fisher’s exact test. For continuous measurements, normality was analyzed using Shapiro–Wilk’s test. Group comparisons were performed with the tests Mann–Whitney U and Kruskal–Wallis H, followed by post hoc analysis using Dunn’s procedure, with a Bonferroni correction for multiple comparisons. The *p* value was considered statistically significant when it was smaller than 0.05.

## 3. Results

The study included a total of 106 male and female patients (teenagers, young adults, and adults) aged between 14 and 60 years, with single-tooth edentulism and a medio–distal dimension between 0 and 4 mm. The patients with spaces between 0 and 1 mm (8.5% from total tooth losses) were treated by closing the edentulous space, while for the rest of the patients the spaces were opened.

**Figure 1:**
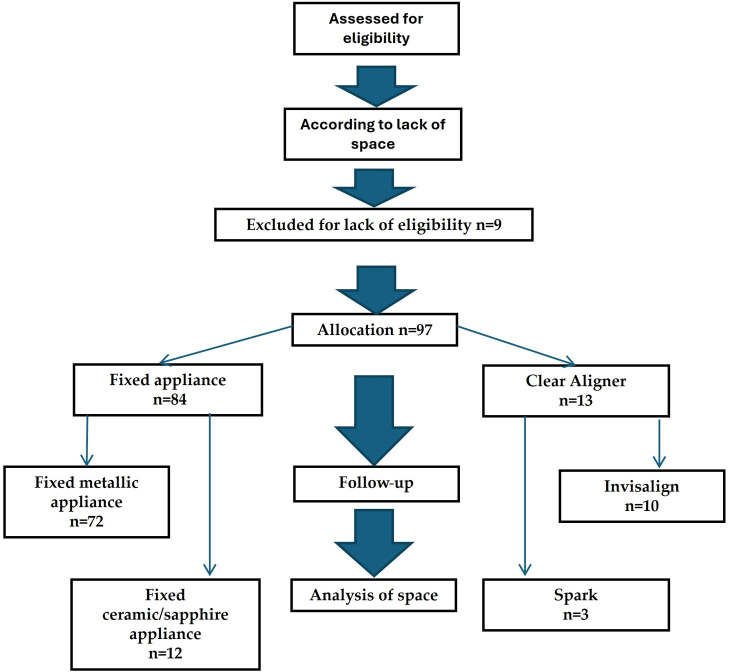
Patients’ distribution in study groups.

Out of the patients treated by opening the edentulous space for implants, 97 (91.5%) were followed and their data were statistically analyzed. The flowchart of patients’ allocation to study groups is presented in Figure 1.

### 3.1. Distribution by Gender of the Study Group

The comparative analysis between genders showed a balanced distribution of clinical and orthodontic characteristics, with no statistically significant differences for most of the parameters analyzed (Table 1). In terms of age groups, there was a predominance of female patients in all categories, especially in the 21–40 age range, but the differences between genders were not significant (*p* = 0.684).

Regarding the distribution of missing teeth, both in the upper and lower arches, the frequencies were comparable between men and women, with higher values in women for lower molars and premolars. As indicated in Table 1, the type of anomalies showed a notable difference between the sexes, with female patients presenting more frequently dental anomalies than males. In terms of the Angle class of malocclusion, the distribution was uniform between the sexes, with a slight predominance of women in all three classes (*p* = 0.573) (Table 2). The position of the interincisal line was also comparable between the two sexes, with most patients having a balanced position of the interincisal line (*p* = 0.737).

The analysis of the relationship between the duration of orthodontic treatment and the gender of patients did not reveal any statistically significant differences (*p* = 0.734). The distribution of treatment durations was similar between men and women, with medium duration treatments (13–24 months) being the most common, followed by those lasting longer than 24 months. Similar proportions between genders suggest that the duration of orthodontic treatment was not significantly influenced by the patient’s gender, but rather by other individual clinical factors. Overall, the results indicated homogeneity between the sexes in terms of age distribution, type of anomalies, Angle class, midline position, and duration of orthodontic treatment, with no major differences between men and women. Analysis of the distribution of potential prosthetic space 1–1.5 years after the start of treatment, according to gender, showed a uniform distribution between the three defined intervals (<6 mm, 6–8 mm, and >8 mm). The values obtained for the statistical tests indicated statistically insignificant differences between male and female patients (*p* > 0.05), suggesting that the evolution of the potential prosthetic space size was not influenced by the patient’s gender.

### 3.2. Distribution by Age of the Study Group

The distribution of the parameters analyzed according to age groups showed a predominance of patients aged between 21 and 40 years (53.6%), followed by those aged between 14 and 20 years (30.9%) and older patients, between 41 and 60 years old (15.5%), with no statistically significant differences between groups (*p* = 0.684) (Table 3). The analysis of missing teeth showed a slight increase in the frequency of tooth loss in the lower molars and premolars compared to the upper ones (mainly first lower molar), but without a significant association with age (*p* = 0.579 for lower molars, *p* = 0.334 for upper molars). The duration of tooth loss did not vary significantly between groups (*p* = 0.654), all groups presenting mainly edentulism of 1–3 years (Table 3).

As described in Table 4, the distribution of Angle class malocclusions varied significantly depending on the age group (*p* = 0.0012). In particular, young patients (14–20 years) presented Angle class II anomalies, reflecting a higher prevalence of sagittal imbalances during adolescence when they may be influenced by craniofacial growth factors. In the 21–40 age group, there was a marked increase in the frequency of Angle class III malocclusions, suggesting either late diagnosis or progressive manifestation of skeletal patterns in this category. In patients over 40 years of age, the distribution is more balanced between classes I and II, with a lower proportion for class III. These results indicated that the type of dental-maxillary anomaly was influenced by age, and the therapeutic approach should be adapted to the specific profile of each age group, given the different evolution of dental-skeletal components throughout life.

The distribution of orthodontic treatment duration according to age does not show statistically significant differences (*p* = 0.317). Medium-length treatments (13–24 months) predominate in all age groups, and the proportion of long treatments (>24 months) increases slightly with age, but without reaching statistical significance. Therefore, the duration of orthodontic treatment does not depend significantly on the patient’s age. Analysis of the distribution of potential prosthetic space size 1–1.5 years after the start of treatment, reported by age group, showed a uniform distribution between the intervals analyzed (<6 mm, 6–8 mm, >8 mm). The Chi-square test (χ^2^) indicated statistically insignificant differences (*p* = 0.742), suggesting that the variation in the size of the potential prosthetic space is not significantly influenced by the age of the patients.

### 3.3. Distribution by Residence of the Studied Parameters

The comparative analysis between the environment of origin (urban and rural) and the orthodontic parameters evaluated did not reveal statistically significant differences for most of the variables included in Table 5. The distribution by age group showed a slight predominance of urban patients in the 21–40 age group, while in rural areas the higher proportion was found in the 14–20 age group; however, the differences were not significant (*p* = 0.149). Regarding missing teeth, the absence of primary molars (M1) was more frequent in rural patients, but no statistical association between the environment of origin and the type of missing teeth was confirmed for either the upper arch (*p* = 0.351) or the lower arch (*p* = 0.144). However, the analysis of the type of anomalies revealed a clear difference: patients from rural areas had significantly more skeletal anomalies, while dental anomalies predominated in urban areas (*p* < 0.0005).

The analysis of the distribution of Angle class of malocclusions according to the environment of origin showed a uniform distribution between urban and rural patients, with no statistically significant differences (*p* = 0.406). Angle malocclusions classes I and II showed almost identical proportions between the patients from the two environments, with approximately two-thirds of patients coming from urban areas, while class III showed a slight urban predominance; however, it was insufficient to significantly influence the overall pattern. These results suggested that the environment of origin does not have a relevant impact on the type of dental-maxillary anomaly according to the Angle classification, the study group presenting a homogeneous epidemiological profile from this point of view. The position of the interincisal line showed a balanced distribution between left and right deviations, with no significant variations between urban and rural areas (*p* = 0.715). Also, the duration of orthodontic treatment was comparable between groups, with most patients requiring between 13 and 24 months of treatment (*p* = 0.712).

Overall, the results suggested that the environment of origin did not significantly influence the clinical and morphological parameters analyzed. The distribution of the potential prosthetic space size 1–1.5 years after the start of treatment, reported according to the environment of origin, did not show statistically significant differences (*p* = 0.379). The results suggested that the urban or rural environment did not influence the evolution of the potential prosthetic space size during the analyzed period.

### 3.4. Baseline Characteristics of the Study Groups

Baseline characteristics of the study groups are presented in the next table (Table 6).

Statistical analysis of the distribution of orthodontic treatment types according to clinical and demographic variables showed that, in general, the differences observed are not statistically significant (*p* > 0.05). In terms of gender distribution, fixed metal appliance treatments predominated in both groups, but the differences between genders were not significant (*p* = 0.398). By age group, young patients (14–20 years) and middle-aged patients (21–40 years) underwent treatment with fixed metal appliances (*p* = 0.525), with no significant differences between age groups.

According to Angle class of malocclusions, fixed metal appliance treatments remained predominant for all three classes (*p* = 0.902), with no relevant variations identified between types of dental-maxillary anomalies (Table 7). Analysis of the interincisal line position showed a slight correlation with the type of treatment, with patients with right deviation having a higher proportion of cases treated with sapphire/ceramic appliances (28.6%) compared to the other groups (*p* = 0.075). In terms of treatment duration, most patients underwent treatment for more than 24 months, with fixed metal appliances predominating (88%), and the differences between the duration groups were not statistically significant (*p* = 0.152). For dental absences, both upper (*p* = 0.450) and lower (*p* = 0.338), no associations were found to significantly correlate between the location of edentulism and the type of orthodontic treatment, although the absence of lower molars (M1) was the most common situation.

Overall, it can be concluded that fixed metal appliance treatments were the most used in all categories analyzed, and the only variable with a statistically significant association with the type of treatment was the position of the interincisal line. The distribution of the size of the potential prosthetic space 1–1.5 years after the start of treatment, as reported according to the type of orthodontic appliance, showed statistically insignificant differences (*p* = 0.149). The results showed that variations in the size of the potential prosthetic space were not significantly influenced by the type of device used.

### 3.5. Distribution of the Studied Parameters According to Edentulous Space Location

A significant association was found between Angle-type anomalies and the edentulous area (*p* = 0.008) (Table 8). In the analyzed group, Angle class II and Angle class III malocclusions were associated with mandibular edentulism, while Angle class I malocclusion occurred more frequently in the maxillary edentulism cases (24 vs. 12).

A significant association was found between the direction of deviation of the interincisal line and the edentulous area, more frequent in the mandible. No statistically significant association was found between the edentulous area and the size of the potential prosthetic space after 1–1.5 years of orthodontic treatment (*p* = 0.546).

Higher space values (>8 mm) were more common in the mandible, while average values (6–8 mm) predominated in the maxillary area.

### 3.6. Correlations Between Studied Parameters and the Duration of Orthodontic Treatment

The correlations between the orthodontic treatment time, the duration of edentulism and the size of the edentulous space were centralized in Table 9.

The analysis of the relationship between the duration of edentulism and the duration of orthodontic treatment revealed statistically significant differences. Patients with recent edentulism, up to 1 year, required orthodontic treatments, and this treatment had intermediate duration between 13 and 24 months (70.3%), while patients with older edentulism, over three years, had prolonged treatments lasting more than 24 months (66.7%). This association was statistically significant (*p* = 0.009), suggesting that as the duration of edentulism increased, the complexity and duration of orthodontic treatment tended to increase. The size of the potential prosthetic space 1–1.5 years after the start of treatment showed a tendency toward higher values (>8 mm) in cases with durations >24 months; however, the overall association was not statistically significant in the Chi-square test (*p* = 0.218).

### 3.7. Correlations Between the Studied Parameters on the Evolution of the Edentulous Space Dimension

The analysis of the initial edentulous space indicated no statistically significant differences between patients with different genders, age groups, residences, or Angle classes (Table 10). For the same parameters, the analysis after the study interval of 1.5 years yielded no statistically significant differences on the space obtained after this period. The only parameter showing statistically significant differences between groups was the position of the interincisal line, where the distributions of the median initial space were similar for all groups of patients (with a center line, a line turned to the left, or a line turned to the right), as assessed by visual inspection of a boxplot. Median initial spaces were statistically significantly different between the three groups of patients, χ^2^(2) = 6.593, *p* = 0.037. Subsequently, pairwise comparisons were performed using Dunn’s procedure with a Bonferroni correction for multiple comparisons. This post hoc analysis revealed no statistically significant differences in median initial spaces between any combination of these three groups. Similar results were obtained for the final edentulous space after 1.5 years (Table 10), χ^2^(2) = 7.881, *p* = 0.019. Subsequently, pairwise comparisons were performed using Dunn’s procedure with a Bonferroni correction for multiple comparisons, and the adjusted *p*-values have been presented. This post hoc analysis revealed statistically significant differences in median initial spaces between the center line group (7.00) and line turned to the right group (5.00) (*p* = 0.040), but not between other group combinations.

Since the edentulous spaces evolved similarly for all patients, regardless of the position of their line, the space variation was similar for all three groups, with no statistically significant differences (*p* = 0.681).

For the patients included in the study lot, aligners were mostly used for patients with a higher initial edentulous space, then a median of approximately 3 mm, while fixed appliances were used for smaller edentulous spaces; thus, a statistically significant difference was identified between the four treatment groups regarding the initial space. χ^2^(3) = 9.187, *p* = 0.027. Subsequently, pairwise comparisons were performed using Dunn’s procedure with a Bonferroni correction for multiple comparisons, and the adjusted *p*-values have been presented. This post hoc analysis revealed statistically significant differences in the median initial spaces between the fixed ceramic group (1.50) and two other groups, the fixed metallic group (3.00) and the Spark group (3.00), *p* < 0.05 for both pairs, but not between other group combinations.

The variation in the edentulous space was slightly higher for aligners, with a median variation of 3.75 mm for Spark and 4.50 mm for Invisalign, compared to a median value of only 3.50 for both types of fixed appliances; still, no statistically significant differences were identified between groups, *p* = 0.315 (Table 11). The final space was also rather different for the three groups, with a median of 7.00 mm for aligners and from 5.00 mm to 6.00 mm for fixed appliances, but with no statistical significance.

The analysis of the treatment type indicated similar median values of the initial edentulous spaces, for aligners and fixed appliances. Spark and fixed metallic appliances were recommended for spaces of around 3 mm, while Invisalign and fixed ceramic appliances were recommended for smaller spaces; therefore, the overall recommendations were balanced between treatment types, with no statistically significant differences between types, *p* = 0.880. The variation in the edentulous space for patients with aligners was slightly higher than the variation for patients with fixed appliances (4.00 mm vs. 3.50 mm), but with no statistically significant differences between groups. Therefore, with similar initial spaces and similar variations, patients ended up with similar final spaces, respecting the trend of the variation: patients with aligners obtained a median final space of 7.00 mm, compared to a median final space of 6.00 mm for patients with fixed appliances, *p* = 0.096.

For patients with fixed appliances, there was a statistically significant difference between those with metallic vs. ceramic/sapphire appliances with respect to the initial edentulous space, as the metallic ones were recommended for larger spaces, while the others were recommended for smaller spaces, *p* = 0.006 (Table 11). The variation was similar for both groups, with an equal median value of 3.5 mm, so there were also statistically significant differences between the final spaces after 1.5 years of treatment, *p* = 0.034 (Table 11).

For patients with clear aligners, no statistically significant differences were identified between patients with Spark and Invisalign for the three studied parameters, *p* > 0.05.

The initial edentulous space also influenced the duration of the orthodontic treatment. Thus, small spaces with median values of 2.00 mm implied a duration of less than a year of orthodontic treatment to achieve the highest final space with a median value of 7.00 mm. Higher spaces with median values of 3.00 mm implied a duration of 2 years or more and may lead to a space of 6.00 mm after the treatment; thus, there were statistically significant differences between the duration of the orthodontic treatment for various dimensions of the initial spaces, χ^2^(2) = 6.878, *p* = 0.032. Pairwise comparisons did not reveal statistically significant differences between any group combinations.

Following this analysis, a multiple regression was run to determine the variation in the edentulous space over time, considering the age of the patient, initial space, position of the interincisal line, type of treatment (aligner of fixed appliance), treatment duration, and edentulism duration. The model was statistically significant F (6, 90) = 2.601, *p* = 0.023, adj. R^2^ = 0.148 (Table 12).

Only the dimension of the initial space was statistically significantly to the edentulous space evolution, *p* = 0.003, as no other parameter significantly contributed to this variation.

## 4. Discussion

Orthodontic treatment can involve significant challenges for clinicians, not only in correcting malocclusion but also in managing patient compliance and behavior. Advances in clear aligner technology have enabled access for an increasing number of patients to more comfortable treatment modalities, with the potential to improve both treatment acceptance and therapeutic outcomes. However, some clinicians have remained cautious regarding the effectiveness of these systems in achieving complex tooth movements [34].

The study determined the effectiveness of orthodontic treatment in creating the potential implant prosthetic space necessary for implant prosthetic restorations in single-tooth edentulism by analyzing fixed orthodontic treatment with different types of brackets (metal, ceramic) and clear aligner treatments. As an effective long-term solution for the replacement of missing teeth, dental implants could have a success rate exceeding 97%, influenced by factors such as age, anatomical location, and edentulous space characteristics [35,36]. Implant restorations in partially edentulous patients could be difficult to achieve because the adjacent teeth tilt or egress, reducing the space needed for inserting an implant. The orthodontic opening of this partially or totally closed space is essential for the prosthetic treatment and for restoring the patients’ masticatory system functions and their aesthetics. The orthodontist must collaborate with the general dentist to establish the dimensions needed for the prosthetic space to ensure the perfect integration of the future implant [26,37].

The current study revealed a significant association between the edentulous area and the size of the potential prosthetic space obtained 1–1.5 years after the initiation of orthodontic treatment. The results suggested that the biomechanical response and remodeling of the edentulous space depended on the location of the arches, with larger space values (>8 mm) tending to be found in the mandible. This can be attributed both to the morpho-functional characteristics of the mandible (increased bone density, greater resistance to orthodontic forces) and to the type of anchorage used. The results were consistent with observations in the literature, which reported greater variability in the spatial response in the mandible compared to the maxilla, depending on the type of appliance and the distribution of the forces applied [38,39,40,41].

The results of the study showed correlations between demographic and morphological parameters and the type of orthodontic treatment applied to prepare the implant prosthetic space. By age group, young patients (12–20 years) and middle-aged patients (21–40 years) accounted for over 80% of the total sample, reflecting an increased demand for orthodontic treatment in younger age patients. This was consistent with most studies showing that adult patients were reluctant to receive orthodontic treatment [42]. On the other hand, age significantly influenced the biological response to orthodontic forces. Adult patients exhibited reduced bone turnover and slower tooth movement, whereas younger patients demonstrated a more active cellular response, leading to faster dental movement [43].

The results regarding the distribution of Angle classes according to age can highlight the existence of distinct patterns of dental-maxillary anomalies during development. The high prevalence of class II in young patients confirmed that anteroposterior imbalances were frequently diagnosed during active growth, when skeletal and functional influences were still dynamic. In contrast, the increased proportion of class III in the 21–40 age group may reflect either a late manifestation of skeletal tendencies or a delayed treatment for this category of anomalies, which are known to have an important genetic component. These results were supported by studies showing that the prevalence of class I appeared to decrease from primary to mixed and permanent dentition, probably due to genetic expression or environmental influences, while Angle’s class II and III remained relatively stable across the three dentitions [44]. In patients over 40 years of age, the balanced distribution suggested stabilization of occlusal patterns and a selection of less severe cases that reach treatment at this age. Overall, these findings have indicated the need to individualize orthodontic treatment plans according to age, as structural dynamics differ significantly between adolescents, young adults, and mature patients.

Treatment plans can include orthodontic space opening or closure before prosthetic therapy using different techniques. Creating sufficient space for implant by fixed or mobile appliances has played a fundamental role in the pre-prosthetic orthodontics [45,46]. Although there have been studies in the literature showing that clear aligners may be a better option for patients with higher periodontal risk or difficulties in maintaining oral hygiene [15], our study showed a majority use of fixed metal appliances, followed by sapphire/ceramic fixed appliances and clear aligners like Invisalign/Spark aligners. Overall, the choice between clear aligners and fixed appliances were guided by individual factors such as age, motivation, and periodontal condition to ensure the best periodontal outcomes.

Konda et al. [47] discussed the efficacy of clear aligners compared to fixed appliances in orthodontic treatment. They concluded that aligners were effective for mild to moderate malocclusions, offering shorter treatments and fewer appointments. According to the present study, both types of appliances were effective in opening the edentulous space for implants; however, fixed metal appliances accounted for the most cases treated, achieving stable results within an average range of 13–24 months. Clear aligner treatments (Invisalign and Spark) were applied to a smaller number of patients with comparable or slightly longer durations, but with moderate correction capacity in cases with extensive edentulous spaces.

When correlating the type of appliance with the size of the edentulous space, fixed appliance treatments—regardless of material—were more effective in restoring narrow spaces (<4 mm) within a moderate time frame (13–24 months), while large spaces (≥4 mm) required longer treatments, regardless of the type of appliance. In conclusion, fixed metal appliances proved to be the most effective in terms of the duration-to-result ratio, offering an optimal balance between space gain stability and total treatment time. Clear aligners can be a viable alternative only in cases with small spaces and increased aesthetic requirements.

This conclusion was not consistent with the results reported by Li et al. [48] and Gaffuri et al. [49], who demonstrated that both Invisalign and fixed appliances can effectively treat complex cases requiring first premolar extractions. However, previous studies reported that Invisalign treatment was 44% longer than treatment with fixed appliances in cases involving the extraction of four premolars [50,51]. This discrepancy may be explained by differences in the treatment protocols used across studies, as well as by variations in patient compliance and the clinician’s level of experience with aligner therapy [42,52].

The duration of orthodontic treatment was influenced by the age of edentulism and, to a lesser extent, by the size of the edentulous space. Recent edentulism (≤1 year) and small spaces (<4 mm) were associated with shorter treatment durations (13–24 months), whereas long-standing edentulism (>3 years) and large spaces (≥4 mm) required longer treatments (>24 months), reflecting increased mechanotherapy complexity. The long-term loss of permanent teeth significantly increased the duration of orthodontic treatment closing the edentulous space, as well as increasing the risk of complications [19].

Midline deviation is a common malocclusion trait, with a similar prevalence in both primary and permanent dentitions, indicating that it does not resolve spontaneously with growth [43]. In this study, the position of the midline did not vary significantly between ages, indicating a uniform occlusal pattern.

It is important to highlight the multidisciplinary aspects of orthodontics and implant treatment involving specialties, such as periodontics, implantology, orthodontics, and prosthodontics; thus, the parameters have gained significant clinical relevance since clinicians must understand the appropriate timing for intervention and the duration required to successfully accomplish the planned therapeutic goals [53,54].

Overall, the results indicated a homogeneity of demographic distributions and an increased prevalence of orthodontic treatment with fixed metal appliances, with the only significant associations relating to the duration of edentulism, the edentulous area, and the type of skeletal anomalies. The gender distribution was balanced with no statistically significant differences in terms of age, the type of anomalies, or the duration of orthodontic treatment. Most treatments lasted between 13 and 24 months, regardless of gender.

Young patients had recent edentulism, while older patients had longer durations of edentulism, which were more difficult to correct orthodontically. The distribution of Angle class malocclusions differed significantly between age groups, highlighting the need to adapt orthodontic strategies according to the patient’s stage of development. The residence influenced the type of anomalies, with skeletal anomalies being more common in patients from rural areas. The position of the interincisal line may have a slight influence on the choice of treatment, with deviations to the right being more commonly associated with sapphire appliances.

Fixed orthodontic appliances have remained the standard for precise tooth movement, while clear aligners have increasingly been used for their aesthetic and periodontal advantages [55,56,57]. Aligners may be less invasive, support better periodontal health, and reduce soft tissue and TMJ-related issues; however, their effectiveness can depend heavily on patient compliance and they are limited in managing complex tooth movements, often requiring a combination with fixed appliances for optimal results [58,59,60].

## 5. Conclusions

In this clinical cohort, the type of orthodontic treatment depended on the patient’s option, with fixed metal appliances being the most used. Angle class II and class III malocclusions were more commonly associated with mandibular edentulism. Orthodontic treatment duration was associated with the duration of edentulism. The edentulous area type and the size of the prosthetic space obtained after 1–1.5 years of orthodontic treatment were correlated. Large edentulous spaces occurred in the mandible, while medium edentulous spaces were more common in the maxillary area, and influenced the clinical decision regarding the treatment plan. Fixed orthodontic appliances and clear aligner therapy were useful in achieving a space opening according to each study group. Early assessments of edentulism and early orthodontic interventions are essential to prevent tooth migration and reduce treatment duration in preparation the prosthetic space for implant.

## Figures and Tables

**Table 1:** Participants’ distribution by gender, age groups, dental abnormalities type and the duration of the orthodontic treatment.

Parameter	Value	Female	Male	Total	*p*
		60 (61.86%)	37 (38.14%)		
Age groups (years old)	14–20	20 (66.7%)	10 (33.3%)	30 (100.0%)	0.684 *
		(33.3%)	(27.0%)		
	21–40	32 (61.5%)	20 (38.5%)	52 (100.0%)	
		(53.3%)	(54.1%)		
	41–60	8 (53.3%)	7 (46.7%)	15 (100.0%)	
		(13.4%)	(18.9%)		
Type of dental abnormalities	Skeletal	12 (48.0%)	13 (52.0%)	25 (100.0%)	0.052 *^#^
		(20.0%)	(35.1%)		
	Dental	48 (66.7%)	24 (33.3%)	72 (100.0%)	
		(80.0%)	(64.9%)		
Duration of the orthodontic treatment	<=12 months	4 (57.1%)	3 (42.9%)	7 (100%)	0.347 **
		(6.7%)	(8.1%)		
	13–24 months	21 (53.8%)	18 (46.2%)	39 (100.0%)	
		(35%)	(48.6%)		
	>24 months	35 (68.6%)	16 (31.4%)	51 (100.0%)	
		(58.3%)	(43.2%)		

* Chi-Square test. ** Fisher’s Exact test. The values in gray are summed by columns, representing the distribution of females and males, according to each parameter described in a line. The total column contains the gender distribution for each category of the parameters described in a line. ^#^ Statistically significant.

**Table 2:** Participants’s dstribution by gender, Angle class, position of the interincisal line and the potential prosthetic space.

Parameter	Value	Female	Male	Total	*p*
		60 (61.86%)	37 (38.14%)		
Angle Class	I	22 (61.1%)	14 (38.9%)	36 (100.0%)	0.573 *
		(36.7%)	(37.8%)		
	II	21 (63.6%)	12 (36.4%)	33 (100.0%)	
		(35.0%)	(32.4%)		
	III	17 (60.7%)	11 (39.3%)	28 (100.0%)	
		(28.3%)	(29.7%)		
Position of the interincisal line	On the center line	21 (60.00%)	14 (40.00%)	35 (100%)	0.737 *
		35.00%	37.84%		
	Turned to the left	20 (58.82%)	14 (41.18%)	34 (100%)	
		33.33%	37.84%		
	Turned to the right	19 (67.86%)	9 (32.14%)	28 (100%)	
		31.67%	24.32%		
Potential prosthetic space size 1–1.5 years after treatment	<6 mm	26 (68.4%)	12 (31.6%)	38 (100.0%)	0.565 *
		(43.3%)	(32.4%)		
	6–8 mm	27 (57.5%)	20 (42.5%)	47 (100.0%)	
		(45%)	(54.1%)		
	>8 mm	7 (58.3%)	5 (41.7%)	12 (100.0%)	
		(11.7%)	(13.5%)		

* Chi-Square test. The values in gray are summed by columns, representing the distribution of females and males, according to each parameter described in a line. The total column contains the gender distribution for each category of the parameters described in a line.

**Table 3:** Participants’ distribution by age groups, absent teeth and the duration of tooth loss.

Parameter	Value	14–20 Years	21–40 Years	41–60 Years	Total	*p*
		30 (30.93%)	52 (53.61%)	15 (15.46%)		
Absent teeth	Upper					0.334 *
	M1	2 (15.4%)	8 (61.5%)	3 (23.1%)	13 (100.0%)	
		(18.2%)	(34.8%)	(60.0%)		
	PM2	2 (18.2%)	8 (72.7%)	1 (9.1%)	11 (100.0%)	
		(18.2%)	(34.8%)	(20.0%)		
	C	1 (100.0%)	0 (0.0%)	0 (0.0%)	1 (100.0%)	
		(9.1%)	(0.0%)	(0.0%)		
	I2	6 (42.9%)	7 (50.0%)	1 (7.1%)	14 (100.0%)	
		(54.5%)	(30.4%)	(20.0%)		
	Lower					
	M1	13 (28.3%)	26 (56.5%)	7 (15.2%)	46 (100.0%)	
		(72.2%)	(89.7%)	(87.5%)		
	PM2	4 (57.1%)	2 (28.6%)	1 (14.3%)	7 (100.0%)	0.579 *
		(22.2%)	(6.9%)	(12.5%)		
	C	1 (50.0%)	1 (50.0%)	0 (0.0%)	2 (100.0%)	
		(5.6%)	(3.4%)	(0.0%)		
Duration of tooth loss	<=1 year	1 (14.3%)	3 (42.9%)	3 (42.9%)	7 (100%)	0.321 *
		(3.3%)	(5.8%)	(20.0%)		
	1–3 years	19 (33.9%)	30 (53.6%)	7 (12.5%)	56 (100%)	
		(63.3%)	(57.7%)	(46.7%)		
	>3 years	10 (29.4%)	19 (55.9%)	5 (14.7%)	34 (100%)	
		(33.3%)	(36.5%)	(33.3%)		

* Fisher’s Exact test. The values in gray are summed by columns, representing the distribution of patients with different age groups, according to each parameter described in a line. The total column contains the age group distribution for each category of the parameters described in a line.

**Table 4:** Distribution by age of the studied parameters.

Parameter	Value	14–20 Years	21–40 Years	41–60 Years	Total	*p*
		30 (30.93%)	52 (53.61%)	15 (15.46%)		
Angle Class	I	10 (27.8%)	20 (55.6%)	6 (16.7%)	36 (100.0%)	0.0012 **^#^
		(33.3%)	(38.5%)	(40.0%)		
	II	17 (51.5%)	10 (30.3%)	6 (18.2%)	33 (100.0%)	
		(56.7%)	(19.2%)	(40.0%)		
	III	3 (10.7%)	22 (78.6%)	3 (10.7%)	28 (100.0%)	
		(10.0%)	(42.3%)	(20.0%)		
Position of the interincisal line	On the center line	11 (31.4%)	17 (48.6%)	7 (20.0%)	35 (100.0%)	0.681 *
		(36.7%)	(32.7%)	(46.7%)		
	Turned to the left	10 (29.4%)	18 (52.9%)	6 (17.6%)	34 (100.0%)	
		(33.3%)	(34.6%)	(40.0%)		
	Turned to the right	9 (32.1%)	17 (60.7%)	2 (7.1%)	28 (100.0%)	
		(30.0%)	(32.7%)	(13.3%)		
Duration of the orthodontic treatment	<=12 months	4 (57.1%)	3 (42.9%)	0 (0.0%)	7 (100.0%)	0.654 **
		(13.3%)	(5.8%)	(0.0%)		
	13–24 months	19 (33.9%)	30 (53.6%)	7 (12.5%)	56 (100%)	
		(63.3%)	(57.7%)	(46.7%)		
	>24 months	10 (29.4%)	19 (55.9%)	5 (14.7%)	34 (100%)	
		(33.3%)	(36.5%)	(33.3%)		
Potential prosthetic space size 1–1.5 years after treatment	<6 mm	10 (26.3%)	21 (55.3%)	7 (18.4%)	38 (100.0%)	0.742 **
		(43.5%)	(37.5%)	(38.9%)		
	6–8 mm	11 (23.4%)	27 (57.4%)	9 (19.1%)	47 (100.0%)	
		(47.8%)	(48.2%)	(50.0%)		
	>8 mm	2 (16.7%)	8 (66.7%)	2 (16.7%)	12 (100.0%)	
		(8.7%)	(14.3%)	(11.1%)		

* Chi-Square test. ** Fisher’s Exact test. ^#^ Statistically significant. The values in gray are summed by columns, representing the distribution of patients with different age groups, according to each parameter described in a line. The total column contains the age group distribution for each category of the parameters described in a line.

**Table 5:** Distribution by residence (urban or rural) of the parameters studied.

Parameter	Value	Urban	Rural	Total	*p*
		68 (70.10%)	29 (29.90%)		
Age groups (years old)	14–20	16 (23.5%)	14 (48.3%)	30 (100.0%)	0.149 *
		(23.5%)	(48.3%)		
	21–40	40 (58.8%)	12 (41.4%)	52 (100.0%)	
		(58.8%)	(41.4%)		
	41–60	12 (80%)	3 (20%)	15 (100.0%)	
		(17.6%)	(10.3%)		
Absent teeth	Upper				0.351 **
	M1	13 (100%)	0 (0.0%)	13 (100.0%)	
		(39.4%)	(0.0%)		
	PM2	9 (81.8%)	2 (18.2%)	11 (100.0%)	
		(24.2%)	(40.0%)		
	C	1 (100%)	0 (0.0%)	1 (100.0%)	
		(3%)	(0.0%)		
	I2	11 (78.6%)	3 (21.4%)	14 (100.0%)	
		(33.3%)	(60.0%)		
	Lower				
	M1	24 (52.2%)	22 (47.8%)	46 (100.0%)	
		(77.4%)	(91.7%)		
	PM2	5 (71.4%)	2 (28.6%)	7 (100.0%)	0.144 **
		(16.1%)	(8.3%)		
	C	2 (100%)	0 (0.0%)	2 (100.0%)	
		(6.5%)	(0.0%)		
Type of dental abnormalities	Skeletal	9 (13.2%)	16 (55.2%)	25 (100.0%)	<0.0005 *^#^
		(13.2%)	(55.2%)		
	Dental	59 (86.8%)	13 (44.8%)	72 (100.0%)	
		(86.8%)	(44.8%)		

* Chi-Square test. ** Fisher’s Exact test. ^#^ Statistically significant. The values in gray are summed by columns, representing the distribution of patients with urban or rural residence, according to each parameter described in a line. The total column contains the residence distribution for each category of the parameters described in a line.

**Table 6:** Participants’ distribution by baseline characteristics.

Parameter	Value	Fixed Metallic Appliance	Fixed Ceramic/ Sapphire Appliance	Invisalign	Spark	Total	*p*
		72 (74.23%)	12 (12.37%)	10 (10.31%)	3 (3.09%)		
	Female	46 (76.7%)	9 (15.0%)	4 (6.7%)	1 (1.7%)	60 (100%)	
Sex		(63.9%)	(75%)	(40%)	(33.3%)		0.398 *
	Male	26 (70.3%)	3 (8.1%)	6 (16.2%)	2 (5.4%)	37 (100%)	
		(36.1%)	(25%)	(60%)	(66.7%)		
	14–20	23 (76.7%)	3 (10.0%)	3 (10.0%)	1 (3.3%)	30 (100%)	
		(31.5%)	(27.3%)	(30%)	(33.3%)		
Age group	21–40	37 (71.2%)	8 (15.4%)	5 (9.6%)	2 (3.8%)	52 (100%)	0.525 **
(years)		(50.7%)	(72.7%)	(50%)	(66.7%)		
	41–60	13 (86.7%)	0 (0.0%)	2 (13.3%)	0 (0.0%)	15 (100%)	
		(17.8%)	(0%)	(20%)	(0%)		
	Upper						
	M1	10 (76.9%)	1 (7.7%)	2 (15.4%)	0 (0.0%)	13 (100%)	
		(13.9%)	(8.3%)	(20.0%)	(0.0%)		
	PM2	8 (72.7%)	2 (18.2%)	1 (9.1%)	0 (0.0%)	11 (100%)	
Absent teeth		(11.1%)	(16.7%)	(10.0%)	(0.0%)		0.450 **
	C	1 (100.0%)	0 (0.0%)	0 (0.0%)	0 (0.0%)	1 (100%)	
		(1.4%)	(0.0%)	(0.0%)	(0.0%)		
	I2	8 (57.1%)	2 (14.3%)	4 (28.6%)	0 (0.0%)	14 (100%)	
		(11.1%)	(16.7%)	(40.0%)	(0.0%)		
	Lower						
	M1	37 (80.4%)	6 (13.0%)	0 (0.0%)	3 (6.5%)	46 (100%)	
		(46.9%)	(40%)	(11.1%)	(100%)		
	PM2	6 (85.7%)	0 (0.0%)	1 (14.3%)	0 (0.0%)	6 (100%)	
		(8.3%)	(0.0%)	(10.0%)	(0.0%)		0.338 **
	C	1 (50.0%)	0 (0.0%)	1 (50.0%)	0 (0.0%)	2 (100%)	
		(1.4%)	(0.0%)	(10.0%)	(0.0%)		
	I2	1 (33.3%)	1 (33.3%)	1 (33.3%)	0 (0.0%)	3 (100%)	
		(1.4%)	(8.3%)	(10.0%)	(0.0%)		

* Chi-Square test. ** Fisher’s Exact test. The values in gray are summed by columns, representing the distribution of patients with various orthodontic treatment types, according to each parameter described in a line. The total column contains the treatment type distribution for each category of the parameters described in a line.

**Table 7:** Distribution of the studied variables in the study groups.

Parameter	Value	Fixed Metallic Appliance	Fixed Ceramic/ Sapphire Appliance	Invisalign	Spark	Total	*p*
		72 (74.23%)	12 (12.37%)	10 (10.31%)	3 (3.09%)		
Angle Class	I	28 (77.8%)	4 (11.1%)	4 (11.1%)	0 (0.0%)	36 (100%)	0.902 *
		(38.4%)	(36.4%)	(40%)	(0%)		
	II	37 (72.5%)	6 (11.8%)	5 (9.8%)	3 (5.9%)	51 (100%)	
		(50.7%)	(54.5%)	(50%)	(100%)		
	III	8 (80.0%)	1 (10.0%)	1 (10.0%)	0 (0.0%)	10 (100%)	
		(11%)	(9.1%)	(10%)	(0%)		
Position of the interincisal line	On the center line	29 (82.9%)	2 (5.7%)	4 (11.4%)	0 (0.0%)	35 (100%)	0.075 *^#^
		(39.7%)	(18.2%)	(40%)	(0%)		
	Turned to the left	26 (76.5%)	1 (2.9%)	4 (11.8%)	3 (8.8%)	34 (100%)	
		(35.6%)	(9.1%)	(40%)	(100%)		
	Turned to the right	18 (64.3%)	8 (28.6%)	2 (7.1%)	0 (0.0%)	28 (100%)	
		(24.7%)	(72.7%)	(20%)	(0%)		
Duration of the orthodontic treatment	<=12 months	5 (71.4%)	1 (14.3%)	1 (14.3%)	0 (0.0%)	7 (100%)	0.152 *
		(6.9%)	(8.3%)	(10%)	(0.0%)		
	13–24 months	47 (73.4%)	9 (14.1%)	6 (9.4%)	2 (3.1%)	64 (100%)	
		(65.3%)	(75.0%)	(60%)	(66.7%)		
	>24 months	20 (76.9%)	2 (7.7%)	3 (11.5%)	1 (3.9%)	26 (100%)	
		(27.8%)	(16.7%)	(30%)	(33.3%)		
Potential prosthetic space size 1–1.5 years after treatment	<6 mm	30 (78.9%)	6 (15.8%)	2 (5.3%)	0 (0.0%)	38 (100%)	0.149 *
		(41.7%)	(50.0%)	(20%)	(0%)		
	6–8 mm	32 (68.1%)	5 (10.6%)	7 (14.9%)	7 (14.9%)	47 (100%)	
		(44.4%)	(41.7%)	(70%)	(100%)		
	>8 mm	10 (83.3%)	1 (8.3%)	1 (8.3%)	0 (0.0%)	12 (100%)	
		(13.9%)	(8.3%)	(10%)	(0%)		

* Chi-Square test. ^#^ Statistically significant. The values in gray are summed by columns, representing the distribution of patients with various orthodontic treatment types, according to each parameter described in a line. The total column contains the treatment type distribution for each category of the parameters described in a line.

**Table 8:** The association between Angle-type anomalies, the direction of interincisal line deviation, and the edentulous area.

Parameter	Value	Maxillary	Mandible	Both	Total	*p*
		43 (44.33%)	53 (54.64%)	1 (1.03%)		
Angle Class	I	24 (66.67%)	12 (33.33%)	0	36 (100%)	0.008 *^#^
		55.81%	22.64%	0%		
	II	14 (42.42%)	18 (54.55%)	1 (1.96%)	33 (100%)	
		32.56%	33.96%	100%		
	III	5 (17.86%)	23 (82.14%)	0	28 (100%)	
		11.63%	43.40%	0%		
Position of the interincisal line	Turned to the feft	6 (17.65%)	27 (79.41%)	1 (2.94%)	34 (100%)	<0.0005 *^#^
		13.95%	50.94%	100%		
	Turned to the right	7 (25.93%)	21 (77.78%)	0	28 (100%)	
		16.28%	39.62%	0%		
	No deviation	30 (85.71%)	5 (14.29%)	0	35 (100%)	
		69.77%	9.43%	0%		
Potential prosthetic space size 1–1.5 years after treatment	<6 mm	14 (36.8%)	23 (60.5%)	1 (2.7%)	38 (100%)	0.546 *
		32.6%	43.4%	100%		
	6–8 mm	23 (48.9%)	24 (51.1%)	0 (0%)	47 (100%	
		53.5%	45.3%	0%		
	>8 mm	6 (50.0%)	6 (50.0%)	0 (0%)	12 (100%)	
		14.0%	11.3%	0%		

* Fisher’s Exact test. ^#^ Statistically significant. The values in gray are summed by columns, representing the distribution of patients with various edentulous area locations, according to each parameter described in a line. The total column contains the edentulous area location distribution for each category of the parameters described in a line.

**Table 9:** Correlations between the duration of orthodontic treatment and the duration of edentulism, the size of the potential prosthetic space 1–1.5 years after treatment, and the size of the edentulous space.

Parameter	Value	≤12 Months	13–24 Months	>24 Months	Total	*p*
		16	48	33		
Duration of the tooth loss	<=1 year	5 (13.5%)	26 (70.3%)	6 (16.2%)	37 (100%)	0.009 **^#^
		(31.3%)	(54.2%)	(18.2%)		
	1–3 years	11 (20.4%)	20 (37.0%)	23 (42.6%)	54 (100%)	
		(68.8%)	(41.7%)	(69.7%)		
	>3 years	0 (0.0%)	2 (33.3%)	4 (66.7%)	6 (100%)	
		(0.0%)	(4.1%)	(12.1%)		
Initial dimension of the edentulous space	<4 mm	16 (19.5%)	41 (50.0%)	25 (30.5%)	82 (100%)	0.086 ^#^*
		(100.0%)	(85.4%)	(75.8%)		
	≥4 mm	0 (0.0%)	7 (46.7%)	8 (53.3%)	15 (100%)	
		(0.0%)	(14.6%)	(24.2%)		
Potential prosthetic space size 1–1.5 years after treatment	<6 mm	3 (8.6%)	18 (51.4%)	17 (40.0%)	38 (100%)	0.218 *
		(42.9%)	(46.2%)	(33.3%)		
	6–8 mm	4 (8.9%)	19 (42.2%)	24 (48.9%)	47 (100%)	
		(57.1%)	(48.7%)	(47.1%)		
	>8 mm	0 (0.0%)	2 (16.7%)	10 (83.3%)	12 (100%)	
		(0%)	(5.1%)	(19.6%)		

* Chi-Square test. ** Fisher’s Exact test. ^#^ Statistically significant. The values in gray are summed by columns, representing the distribution of patients based on the treatment duration, according to each parameter described in a line. The total column contains the treatment duration distribution for each category of the parameters described in a line.

**Table 10:** Variation in the edentulous space (expressed in mm) according to the study group characteristics.

Parameter	Value	Initial Space	Final Space After 1.5 Years	Space Difference
		Median	Median	Median
	Female	2.75	6.00	3.50
Sex	Male	2.50	6.00	4.00
	*p* *	0.384	0.249	0.250
Age group	14–20	2.50	6.00	3.50
	21–40	2.50	6.00	4.00
	41–60	3.00	7.00	4.00
	*p* **	0.697	0.509	0.679
Environment	Urban	2.50	6.00	3.75
	Rural	2.50	5.00	3.50
	*p* *	0.727	0.137	0.350
Angle Class	I	2.50	6.00	3.75
	II	2.50	6.00	4.00
	III	3.000	5.00	3.00
	*p* **	0.424	0.106	0.123
Position of the interincisal line	On the center line	3.00	7.00	4.00
	Turned to the left	2.00	6.00	3.75
	Turned to the right	2.25	5.00	3.50
	*p* **	0.037 ^#^	0.019 ^#^	0.681

* Mann–Whitney U test. ** Kruskal–Wallis H test. ^#^ Statistically significant. The values in grey represent the *p* values for each parameter in a line.

**Table 11:** Variation in the edentulous space (expressed in mm) according to orthodontic treatment type.

Parameter	Value	Initial Space	Final Space After 1.5 Years	Difference
		Median	Median	Median
Treatment	Spark	3.00	7.00	3.75
	Invisalign	2.50	7.00	4.50
	Fixed Metallic	3.00	6.00	3.50
	Fixed Ceramic	1.50	5.00	3.50
	*p* *	0.027 ^#^	0.063	0.315
Treatment type	Aligners	2.50	7.00	4.00
	Fixed appliances	2.50	6.00	3.50
	*p* **	0.880	0.096	0.111
Fixed appliances	Metallic	3.00	6.00	3.50
	Ceramic/sapphire	1.50	5.00	3.50
	*p* **	0.006 ^#^	0.034 ^#^	0.563
Clear Aligners	Spark	3.00	7.00	3.75
	Invisalign	2.50	7.00	4.50
	*p* **	0.260	0.825	0.330
Duration of the orthodontic treatment	<=12 months	2.00	7.00	5.00
	13–24 months	2.50	6.00	4.00
	>24 months	3.00	6.00	3.50
	*p* *	0.032 ^#^	0.396	0.411

* Kruskal–Wallis H test. ** Mann–Whitney U test. ^#^ Statistically significant. The values in grey represent the *p* values for each parameter in a line.

**Table 12:** Multiple regression results in the variation in the edentulous space over time.

Space Variation	B	95% CI for B		Se B	β	Sig
		Low Limit	Upper Limit			
Age	0.025	−0.006	0.055	0.015	0.169	0.113
Initial space	−0.481	−0.795	−0.167	0.158	−0.318	0.003 ^#^
Interincisal line	−0.324	−0.766	0.118	0.222	−0.156	0.149
Treatment type	0.577	−0.446	1.600	0.515	0.118	0.266
Treatment duration	−0.197	−0.594	0.200	0.200	−0.106	0.327
Edentulism duration	0.080	−0.495	0.656	0.290	0.030	0.782

^#^ Statistically significant.

## Data Availability

The data presented in this study are available on request from the corresponding author due to privacy, legal, and ethical restrictions.
